# MRBLES 2.0: High-throughput generation of chemically functionalized spectrally and magnetically encoded hydrogel beads using a simple single-layer microfluidic device

**DOI:** 10.1038/s41378-020-00220-3

**Published:** 2020-11-30

**Authors:** Yinnian Feng, Adam K. White, Jamin B. Hein, Eric A. Appel, Polly M. Fordyce

**Affiliations:** 1 Department of Genetics, Stanford University, Stanford, CA 94305 USA; 2 Department of Bioengineering, Stanford University, Stanford, CA 94305 USA; 3 Department of Biology, Stanford University, Stanford, CA 94305 USA; 4 The Novo Nordisk Foundation Center for Protein Research, Faculty of Health and Medical Sciences, University of Copenhagen, Blegdamsvej 3b, 2200 Copenhagen, Denmark; 5 Department of Materials Science & Engineering, Stanford University, Stanford, CA 94305 USA; 6 Stanford ChEM-H, Stanford University, Stanford, CA 94305 USA; 7 Chan Zuckerberg Biohub, San Francisco, CA 94110 USA

**Keywords:** Nanoscience and technology, Chemistry, Engineering, Materials science

## Abstract

The widespread adoption of bead-based multiplexed bioassays requires the ability to easily synthesize encoded microspheres and conjugate analytes of interest to their surface. Here, we present a simple method (MRBLEs 2.0) for the efficient high-throughput generation of microspheres with ratiometric barcode lanthanide encoding (MRBLEs) that bear functional groups for downstream surface bioconjugation. Bead production in MRBLEs 2.0 relies on the manual mixing of lanthanide/polymer mixtures (each of which comprises a unique spectral code) followed by droplet generation using single-layer, parallel flow-focusing devices and the off-chip batch polymerization of droplets into beads. To streamline downstream analyte coupling, MRBLEs 2.0 crosslinks copolymers bearing functional groups on the bead surface during bead generation. Using the MRBLEs 2.0 pipeline, we generate monodisperse MRBLEs containing 48 distinct well-resolved spectral codes with high throughput (>150,000/min and can be boosted to 450,000/min). We further demonstrate the efficient conjugation of oligonucleotides and entire proteins to carboxyl MRBLEs and of biotin to amino MRBLEs. Finally, we show that MRBLEs can also be magnetized via the simultaneous incorporation of magnetic nanoparticles with only a minor decrease in the potential code space. With the advantages of dramatically simplified device fabrication, elimination of the need for custom-made equipment, and the ability to produce spectrally and magnetically encoded beads with direct surface functionalization with high throughput, MRBLEs 2.0 can be directly applied by many labs towards a wide variety of downstream assays, from basic biology to diagnostics and other translational research.

## Microfluidics: high-throughput production of spectrally encoded hydrogel beads for bioassays

Spectrally encoded beads provide a convenient platform for multiplexed bioassays, offering fast binding kinetics and many replicates per assay. A recently developed technology, MRBLEs, spectrally encodes hydrogel beads via the ratiometric incorporation of lanthanide nanophosphors. In the present paper, a team from Stanford University led by Polly Fordyce reports a dramatically simplified method for producing MRBLEs beads bearing various functional groups for downstream chemical coupling or on-bead synthesis. Using a ‘jumper cable’ tubing strategy, they route microfluidic channels in 3D without a need for complex fabrication techniques to create multi-nozzle droplet generators. Using these simple single-layer microfluidic devices, they create beads with 48 unique spectral codes bearing carboxyl and amine groups for downstream coupling with over 1000-fold increase in throughput. Finally, they demonstrate that MRBLEs can be simultaneously spectrally and magnetically encoded.

## Introduction

The recently developed ability to identify molecules across a cell’s genome, transcriptome, and proteome has dramatically increased the need for technologies capable of detecting interactions between biological macromolecules at scale to understand interactome networks. Multiplexed bioassays, in which binding is assessed between a single ‘bait’ molecule and many possible “prey” interactors, can reduce the number of experiments required to explore a potential interactome space and thus speed the pace of discovery. Pioneering examples of these assays used spatial arrays to test thousands to millions of potential interactions in a single experiment^1,2^. However, spatial arrays suffer from relatively slow kinetics (as bait molecules are immobilized on a planar surface) and typically require relatively large amounts of sample^1^.

Multiplexed bead-based assays provide an appealing alternative, with near fluid-phase interfacial kinetics, many replicates per experiment, opportunities for quality control, and the ability to flexibly couple different probes and targets across experiments^3–5^. Spectrally encoded beads, which are embedded with ratiometric combinations of fluorescent (e.g., organic dyes^6–8^, quantum dots (QDs)^9–12^) or luminescent materials (e.g., lanthanide nanoparticles^13–15^), provide a particularly convenient format for multiplexed assays and have already been used for a wide variety of applications^15–25^. To date, Luminex multianalyte profiling (xMAP) technology represents the most widely used spectrally encoded bead-based technology. Luminex xMAP beads are commercially available, are compatible with flow cytometry^26^ and are widely used for a variety of bioassays^27^. These beads consist of magnetic polystyrene microspheres that encapsulate distinct proportions of red and infrared fluorophores, each of which comprises a unique spectral code. However, these beads also suffer from a variety of limitations. First, the use of fluorescent dyes for encoding limits the possible coding space to 500, and code sets typically contain <100 codes. Second, hydrophobic polystyrene beads cannot detect low-affinity interactions due to widespread nonspecific binding mediated by hydrophobic interactions^28^.

Recently developed spectrally encoded hydrogel beads provide an appealing alternative that has been used for a variety of biomedical and sensing applications^29,30^. Hydrogel beads are comprised of a cross-linked, hydrated polymeric network made up of one or more hydrophilic monomers, providing near fluid‐phase kinetics at functionalized surfaces as well as high-efficiency molecular loading^31,32^, and microfluidic droplet generators have been used to produce and polymerize hydrogel beads with high throughput^33^. However, the use of fluorescent organic dyes to create spectral codes in most cases has limited the number of unique spectral codes to <100^7,8^. Moreover, such encoded microspheres are often incompatible with harsh organic solvents, precluding their use in a variety of solid-phase synthesis applications. While QD-based spectrally encoded hydrogel beads have been well established^9–12^, QD-based encoding suffers from potential chemical instability, short luminescence lifetimes, and photoblinking due to their complicated photophysics^34,35^. In addition, tightly packed QDs inside the bead matrix can undergo energy transfer and reabsorption^36^. As a result, the demonstrated code spaces for QD-based approaches have been limited to ≤10, far short of the number in theoretical simulations. Furthermore, concerns about potential biotoxicity can hinder their broad application.

Our group recently developed a novel technology (MRBLEs, for microspheres with ratiometric barcode lanthanide encoding) that spectrally encodes beads via the ratiometric incorporation of lanthanide nanophosphors (Lns)^14^. Lns have narrow and well-separated emission spectra, making it theoretically possible to generate code sets with 10^5^-10^6^ unique members^13^, and we previously demonstrated the ability to produce and discriminate >1100 codes^14^. In addition, MRBLEs can be subsequently functionalized for on-bead solid-phase peptide synthesis^37,38^. However, prior MRBLE production pipelines were complex, requiring two-layer microfluidic devices with integrated valves and extensive custom pneumatics control hardware^13,14^, limiting their widespread adoption. In addition, the production throughput was slow (requiring 1.5 h to produce ~10,000 beads with a given embedded code), making bead generation for assay development inefficient and labor intensive.

Here, we present a simple method (MRBLEs 2.0) for the efficient high-throughput generation of spectrally encoded and magnetic beads bearing various functional groups capable of downstream chemical coupling or on-bead synthesis. MRBLEs 2.0 relies on the manual mixing of Ln/polymer mixtures followed by droplet generation using single-layer, parallel flow-focusing (FF) devices and off-chip batch polymerization of droplets into beads, thereby simplifying and enhancing the throughput of bead production. To facilitate downstream analyte coupling, MRBLEs 2.0 localizes copolymers bearing functional groups typically used for bioconjugation to the surface of the hydrogel matrix during droplet generation and covalently crosslinks these polymers in place during bead polymerization. Using the MRBLEs 2.0 pipeline, we demonstrate the ability to generate monodisperse MRBLEs (CV < 7%) containing 48 distinct well-resolved spectral codes (<0.01% probability of code misassignment) with high throughput (>~3,000,000 beads with diameter ~50 μm within 20 min per code, including off-chip polymerization and washing steps). To highlight the utility of these MRBLEs in downstream assays, we conjugate amine-functionalized oligonucleotides and entire proteins to MRBLEs bearing carboxyl groups with estimated bead loading densities of 10^7^–10^8^ molecules/bead and additionally conjugate biotin molecules to amine-functionalized MRBLEs, demonstrating the feasibility of direct on-bead peptide synthesis^37,38^. To facilitate bead separation and washing during assays, we additionally show that spectrally encoded beads can also be magnetized via the simultaneous incorporation of magnetic nanoparticles with little effect on spectral codes. Finally, for potential assays requiring large numbers of beads, we use droplet splitting to exponentially amplify bead production within the same microfluidic chip footprint. We anticipate that MRBLEs 2.0 will be a broadly useful pipeline for a variety of bioassays designed to detect DNA hybridization, identify protein-protein/peptide interactions, and screen polymers for useful bioactivity. Furthermore, this MRBLEs 2.0 production pipeline requires easy-to-fabricate single-layer PDMS devices and no custom-made equipment. Therefore, we anticipate that this simple technology for high-throughput encoded bead production can be easily adopted by other labs and companies.

## Results

### High-throughput production of MRBLEs using a novel parallel flow focuser and syringe pumps

MRBLEs 2.0 requires only 2 syringe pumps, a low-cost microscope, a flood UV illumination source, and an easily fabricated single-layer microfluidic device. To allow parallel microfluidic droplet generation, the device uses PEEK tubing as ‘jumper cables’ to provide three-dimensional routing of flow channels (see Figure S1 and Supplemental Methods) without the need for complex multilayer PDMS device fabrication or laser ablation to create connections between layers^39^.

**Fig. 1:**
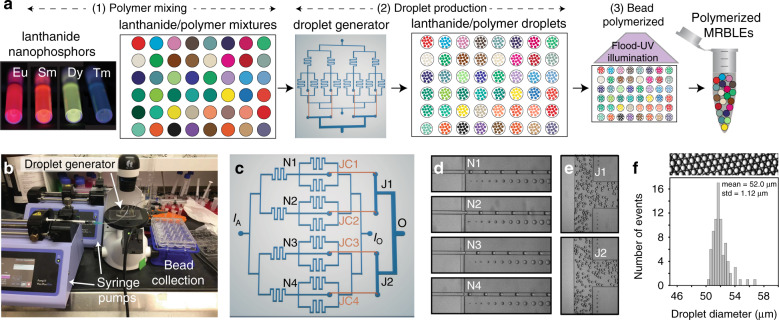
MRBLEs 2.0 high-throughput bead production. **a** Overall experimental pipeline: (1) ‘polymer mixing’: ratiometric combinations of lanthanide nanophosphors (each of which comprises a unique spectral code) are mixed with aqueous polymer in individual wells of a multiwell plate; (2) droplet production: mixtures are iteratively introduced into a single-layer microfluidic droplet generation device; (3) bead polymerization: droplets corresponding to each code are collected after synthesis and are exposed to UV light to drive polymerization into solid beads. **b** Low-cost setup for high-throughput MRBLE synthesis consisting of 2 syringe pumps and an inexpensive inverted microscope. **c** Schematic of a single-layer microfluidic device. Aqueous polymer and oil solutions are introduced at *I*_A_ and *I*_O_ and meet at 4 flow-focusing nozzles (N1-4); produced droplets are routed from four junctions to two additional junctions (J1-2) via ‘jumper cable’ tubing (JC1-4) prior to collection from the droplet outlet (O). **d** Images of droplet generation at the 4 flow-focusing nozzles (N1-4). **e** Images of droplets merging at junctions J1 and J2 after passing through ‘jumper cables’. **f** Representative image (top) and histogram (bottom) showing measured diameters for produced droplets (diameter = 52.0 ± 1.12 µm, CV = 2%, 79 droplets)

The pipeline produces MRBLEs in 3 stages, as shown in Fig. 1a: (1) off-chip generation of Ln/polymer mixtures that will ultimately comprise MRBLEs (“polymer mixing”), (2) on-chip production of Ln/polymer droplets using parallel microfluidic flow focusers (“droplet production”), and (3) off-chip polymerization of Ln/polymer droplets into solid MRBLEs via exposure to UV light (“bead polymerization”). In the first stage (“polymer mixing”), ratiometric mixtures of polyacrylic acid (PAA)-wrapped Lns (typically 24, 48, or 96 distinct mixtures, each corresponding to a unique spectral code) are combined with a polymer solution off-chip via manual or robotic pipetting and deposited into a standard multiwell plate. At this point, polymer solutions can be stored at 4 °C in the dark for weeks prior to MRBLE synthesis. Immediately before MRBLE droplet production, a UV-activated photoinitiator (lithium phenyl-2,4,6-trimethylbenzoylphosphinate, LAP) is added to each encoded polymer solution to facilitate subsequent off-chip bead polymerization.

In the second stage (“droplet production”), these Ln/polymer mixtures and a fluorinated oil solution (HFE7500 + 2% w/w Ionic Krytox surfactant) are simultaneously introduced into a microfluidic droplet generator using syringe pumps at specific volumetric flow rates (Fig. 1b**)**. Within the device, these aqueous polymer and fluorinated oil streams meet at four parallel droplet generation nozzles, where changes in interfacial tension cause the aqueous polymer to break off and form monodisperse droplets within the oil stream (Fig. 1c, d; Movie S1). “Jumper cables” route droplets produced at each nozzle to two junctions (Fig. 1c–e) and subsequently to a single output, enhancing production rates 4-fold while allowing the collection of all droplets containing a given code within a single well of the 24-well plate. Droplet sizes can be tuned by simply varying the flow rates of aqueous and oil phases (Fig. S2). Here, we used flow rates of 600 µL/h (aqueous) and 3200 µL/h (oil) to generate stable droplets with a measured median diameter of ~52 µm and an overall CV of 2% (Fig. 1f). Between codes, the entire device can be flushed with continuously running oil, and the aqueous tubing (Fig. S1b, assembled in step 1) can be flushed with water to prevent cross-contamination.

**Fig. 2:**
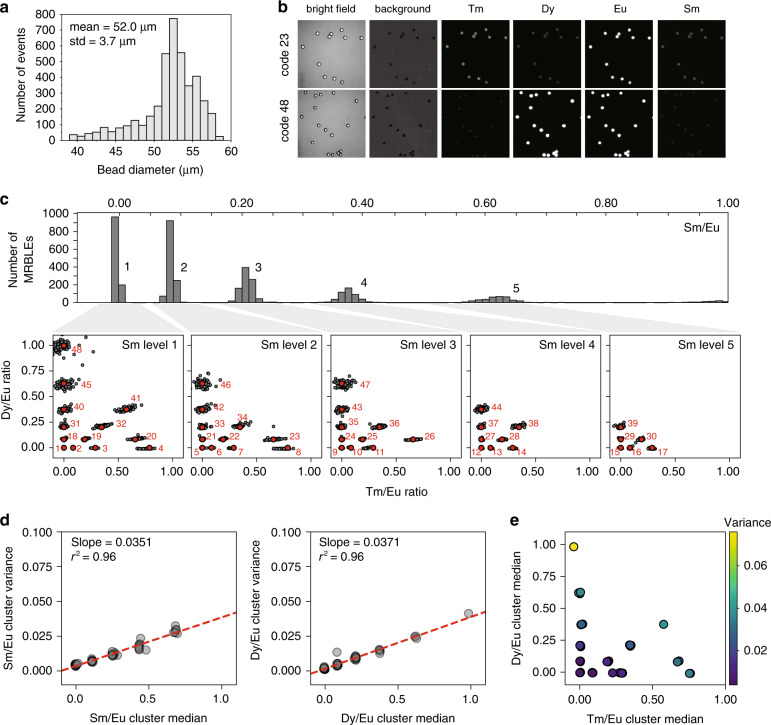
MRBLEs 2.0 spectral codes and code cluster characterization. **a** Histogram showing measured diameters for polymerized MRBLEs (diameter = 52.0 ± 3.7 µm, CV = 7%, 4138 beads). **b** Representative bright-field, unmixed background and Lns emission images after the linear unmixing of lanthanide emission images of MRBLEs 2.0 beads for two different example spectral codes (23 and 48). **c** Histogram of Sm bead intensities (top) and scatter plots showing measured Dy/Eu and Tm/Eu ratios for each Sm intensity level (bottom) for 48 code clusters (4138 beads in total). **d** Sm/Eu (left) and Dy/Eu (right) cluster variance as a function of the cluster median. **e** Tm cluster variance as a function of the Dy/Eu and Tm/Eu cluster medians

In the final stage (“bead polymerization”), all droplets from a given code are transferred to a single well of a multiwell plate and the entire plate is exposed to flood UV illumination, allowing the simultaneous polymerization of all droplets into solid beads. This pipeline typically produces 3,000,000 MRBLEs/20 min, enhancing throughput by >1000-fold compared to prior production methods^14^. Polymerized MRBLEs remain highly monodisperse, with a measured median diameter of 52 µm and an overall CV of 7% (Fig. 2a and Fig. S3). This CV is slightly higher than that seen with prior lower-throughput production methods (7% vs 5%, respectively), likely due to a combination of oxygen inhibition and overheating that can induce droplet breakage during off-chip polymerization. However, the benefits associated with enhanced throughput likely outweigh the deleterious effects of a small increase in CV for all but the most sensitive applications.

### Spectral codes within MRBLEs produced with high throughput remain easily resolved from one another

Using MRBLEs in downstream multiplexed assays requires the ability to distinguish embedded codes from one another with high confidence. To test the robustness of spectral encoding and determine the maximum likely coding capacity achievable with high-throughput production, we designed a target matrix of 48 distinct codes comprised of ratios of dysprosium (Dy), samarium (Sm), thulium (Tm), and europium (Eu) Lns (Dy/Eu, Sm/Eu, and Tm/Eu). We then mixed these ratios off-chip via simple manual pipetting, generated droplets from these mixtures, polymerized the droplets, washed extensively with solvents (to remove unpolymerized material), and imaged the resultant MRBLEs to quantify observed Ln intensity ratios and compare these measured ratios to the desired target ratios.

Images of MRBLEs excited in the deep UV (292 nm) with emissions collected across 9 Ln emission channels (435, 474, 536, 546, 572, 620, 630, 650, and 780 nm) established that beads composed of a 21.4% v/v poly(ethylene glycol) diacrylate (PEG-DA) matrix were homogeneously polymerized after UV flood exposure without detectable Ln aggregation (Figure S4**)**. Ln intensities can be extracted after linear unmixing using the normalized spectral reference of each Ln across all Ln emission channels and can be assigned to specific codes (Fig. 2b). However, we also observed that higher concentrations of PEG-DA led to dramatic aggregation of Lns (Fig. S5)^14^, likely due to PEG-DA absorption of water molecules required to maintain charge-charge repulsion of polyacrylic acid-wrapped Lns.

In prior work, we observed that emission spectra for Sm are largely orthogonal to those of other Lns such that observed Sm/Eu intensities depend only on the amount of Sm incorporated within each bead^13,14^. Consistent with this, we observed five clearly separable Sm/Eu ratios that corresponded directly to the intended Sm/Eu target levels (Fig. 2c, top). By considering only beads at a given Sm/Eu ratio and plotting the observed Dy/Eu ratio against the observed Tm/Eu ratio for each bead, we can further directly visualize individual code clusters and compare each cluster to its intended target value (Fig. 2c, bottom). All 48 code clusters are present and easily distinguished from one another with the median observed cluster intensity ratio well centered on the desired target. For Sm/Eu and Dy/Eu ratios, the observed cluster variance depended linearly on the median Sm/Eu or Dy/Eu ratio, respectively (Fig. 2d), with a slope of ~0.04 for both Lns. By contrast, the variance for clusters in the Tm/Eu channel depended on both the median Tm/Eu and Dy/Eu ratios (Fig. 2e), reflecting the fact that Dy and Tm both emit light in the 474 nm emission channel, contributing to crosstalk (Fig. S6a, b). This measured cluster variance as a function of median value suggests that Sm, Dy, Tm, and Eu could be used to produce well-resolved code sets with 208 clusters separated by five standard deviations between each cluster or even larger code sets with smaller separations (839 or 380 clusters at 3 and 4 standard deviation separations, respectively) (Fig. S6c).

### Direct MRBLE functionalization during off-chip polymerization

**Fig. 3:**
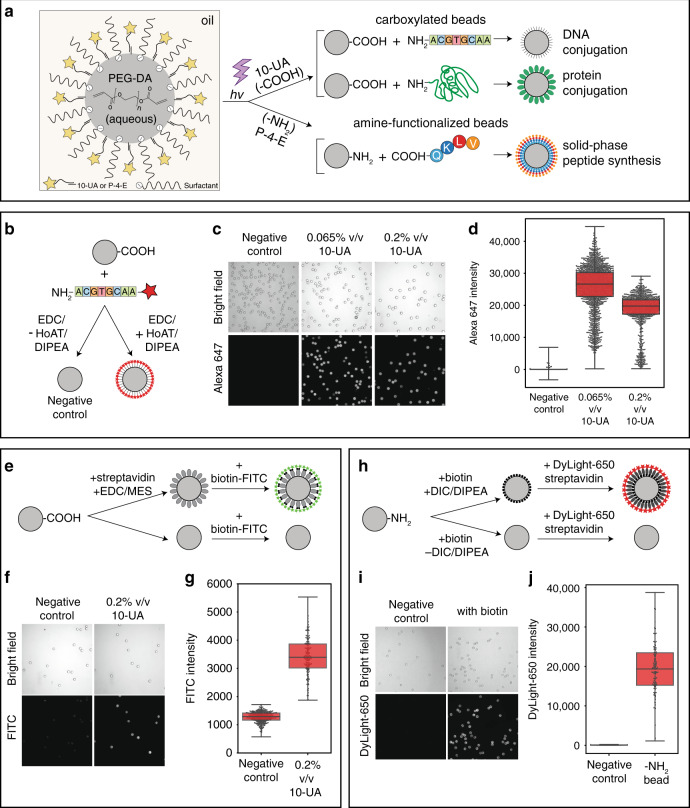
Covalent conjugation of oligonucleotides, proteins, and biotin to MRBLEs copolymerized with functionalized monomers. **a** Schematic illustrating the functionalization of PEG-DA hydrogel beads for downstream DNA or protein coupling or on-bead peptide synthesis. **b** Assay used to quantify the specific coupling of fluorescently labeled amine-functionalized oligonucleotides to carboxylated beads. **c** Bright-field (top) and fluorescence (bottom) images of MRBLEs polymerized in the presence of 0.065% or 0.2% v/v 10-UA comonomers after conjugation with and without required coupling reagents (negative control beads were polymerized with 0.2% v/v 10-UA comonomers). **d** Median Alexa 647 intensities for all MRBLEs from each condition. **e** Assay used to quantify coupling of streptavidin proteins to carboxylated beads. **f** Representative images of MRBLEs incubated with biotin-FITC. **g** Median FITC intensities for all MRBLEs from each condition. **h** Assay used to quantify on-bead peptide synthesis. **i** Representative images of MRBLEs after incubation with DyLight 650-labeled streptavidin. **j** Median DyLight 650 intensities for all MRBLEs from each condition. Note that all conjugation experiments were performed using code 1

Multiplexed bioassays designed to screen for binding interactions require the ability to couple ‘bait’ analytes of interest (e.g., DNA oligonucleotides, whole proteins, or chemically synthesized peptides) to the MRBLE surface at high density and without crosstalk or analyte dissociation over long storage timescales. Covalent coupling is particularly robust and typically relies on two main functional groups: -COOH (carboxyl groups) and -NH_2_ (amine groups) (Fig. 3a). Carboxyl groups on the bead are compatible with 1-ethyl-3-(3-dimethylaminopropyl) carbodiimide (EDC) chemistry, which facilitates subsequent covalent coupling of any molecules bearing a free amine group to beads in aqueous/organic solvents (e.g., whole proteins bearing an exposed primary amine or amine-functionalized DNA oligonucleotides). Conversely, amine groups displayed on MRBLEs provide convenient handles for the covalent coupling of carboxylated molecules, such as the solid-phase synthesis of peptides directly on beads via standard Fmoc coupling chemistry.

In prior work, –COOH or –NH_2_ moieties were covalently coupled to the PEG-DA polymer chains comprising the MRBLE hydrogel matrix via Michael addition after bead synthesis^38^. However, this method was relatively slow (requiring ~24–48 h) and susceptible to the inhomogeneous distribution of free acrylate groups on the bead surface, leading to heterogeneity of coverage during coupling. To address these problems, we developed a novel functionalization approach that leverages solubility differences between –COOH– or –NH_2_-terminated comonomers and the photoinitiator (LAP) to drive the polymerization of comonomers at the surface of the hydrogel matrix only (Fig. 3a). The comonomers used here (10-undecenoic acid (10-UA) for -COOH and pent-4-enylamine (P-4-E) for -NH_2_) are soluble in HFE 7500 oil, while LAP is only soluble in water. The exposure of LAP-containing aqueous droplets to UV drives a polymerization chain reaction that begins in the aqueous PEG-DA microsphere core and ultimately reaches the oil/water interface to drive the cross-linking of oil-soluble interfacial comonomers to the hydrogel bead matrix (Fig. 3a). As the concentration of comonomer within the bulk oil phase is very low (0.2% v/v for 10-UA and 0.09% v/v for P-4-E), the chain reaction is then terminated to limit cross-linking to this oil/water interface.

### Direct coupling of oligonucleotides and proteins to MRBLEs and on-MRBLE biotin conjugation

To demonstrate this technique and quantify coupling efficiency and variability for DNA conjugation, we synthesized MRBLEs bearing -COOH functional groups at the surfaces and directly coupled fluorescently labeled (Alexa 647), NH_2_-functionalized oligonucleotides to them via EDC chemistry (Fig. 3b). Images of beads after coupling and washing established that the fluorescence intensities are relatively even across beads (CV ~27%) and high only when coupling is performed in the presence of HoAT/DIPEA coupling reagents, establishing that coupling is specific (Fig. 3b–d). Bright-field and fluorescence images further establish that beads are undamaged after exposure to coupling reagents and that unbound oligonucleotides are effectively removed by washing (Fig. 3b–d). The comparison of fluorescence signals (Alexa 647) of beads (both using code 1) polymerized in the presence of two different concentrations of 10-UA revealed higher but slightly more heterogeneous intensities for 0.065% (v/v) 10-UA. To eliminate any batch-to-batch variation from separate conjugation and imaging, we additionally conjugated the same oligonucleotide simultaneously to two different codes bearing different percentages of added 10-UA (code 1 with 0.065% v/v 10-U and code 18 with 0.02% v/v 10-UA, as shown in Fig. S7) and imaged them together. Again, the 0.065% v/v 10-UA condition led to stronger fluorescence intensities than the 0.2% v/v 10-UA condition with a smaller variance in intensities. These observations were again consistent with a model in which the incorporation of –COOH groups on the bead surface is more homogenous at higher 10-UA concentrations^40^. The estimation of loading densities by DNA conjugation for the 0.2% (v/v) 10-UA condition suggested that ~10^7^ to 10^8^ oligos bind to the surface of each bead, consistent with prior estimates of MRBLE peptide loading capacities^38^.

To demonstrate that carboxylated MRBLEs can also be used for the covalent coupling of entire proteins under aqueous conditions, we tested the ability to attach streptavidin molecules to MRBLE surfaces via their primary amines. To visualize the attached streptavidin, we then incubated beads with biotin-FITC conjugates, washed them three times and imaged them (Fig. 3e). As with the DNA conjugation reactions, we observed significant FITC intensities only after covalently coupling streptavidin to surfaces and not in the absence of coupling reagents (Fig. 3f, g).

Finally, we demonstrated the feasibility of using NH_2_-functionalized MRBLEs for solid-phase peptide synthesis by (1) polymerizing MRBLEs with P-4-E (0.09%, v/v) added to the oil phase, (2) incubating these functionalized MRBLEs with biotin (which bears a free –COOH group analogous to standard Fmoc-amino acids) in either the presence or absence of required coupling reagents, (3) incubating with DyLight 650-labeled streptavidin, (4) washing, and then (5) imaging to quantify the amount of bead-bound fluorescence (Fig. 3h). As with the other coupling reactions, we observed strong fluorescence only in the presence of coupling reagents, establishing that the presented NH_2_ groups can be used for subsequent specific conjugation (Fig. 3i, j).

### Spectrally encoded magnetic MRBLEs improve separation efficiencies and increase coding capacity

The ability to selectively magnetize particles provides an additional coding axis, thereby increasing the number of codes that can be distinguished from one another (e.g., generating the same set of spectral codes in the presence and absence of magnetic particles increases coding capacity 2-fold). In addition, magnetic beads have particular advantages for bead-based separation, improving bead retention during rinsing and the removal of excess supernatant via the application of a magnetic field and facilitating more stringent washing. Finally, magnetic beads can also aid in loading beads into microwells for high-throughput experiments (e.g., single-cell phenotyping).

**Fig. 4:**
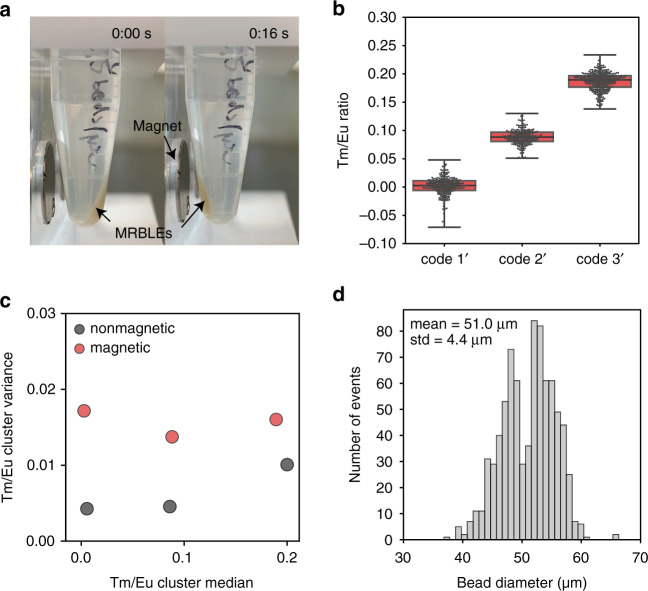
MRBLEs 2.0 beads containing embedded spectral and magnetic codes. **a** Images showing magnet-induced movement of MRBLEs 2.0 beads containing magnetic nanoparticles. **b** Measured Tm/Eu intensities for MRBLEs containing magnetic nanoparticles and three different closely spaced Tm/Eu ratios. Note that the three Tm/Eu ratios are codes 1, 2, and 19 from the original 48 code sets. **c** Measured cluster variance of Tm/Eu code clusters in the presence and absence of magnetic nanoparticles. **d** Measured size distribution of spectrally encoded magnetic MRBLEs (*n* = 812)

To test the ability to create magnetic MRBLEs, we synthesized Fe_3_O_4_ nanoparticles by modifying a previously published coprecipitation method^41^ to include extra PAA in the precursor solution, thereby enhancing the control of nucleation and nanoparticle wrapping as well as preventing nanoparticle aggregation. PAA wrapping also prevents potential reductions in luminescence from Lns caused by direct contact with magnetic nanoparticles^42^, as the thickness^43^ and electrostatic repulsion of PAA increase interparticle distances beyond those that lead to quenching^42^. The resultant Fe_3_O_4_ nanoparticles were ~46 nm in diameter and appeared optically transparent (Fig. S8). When these Fe_3_O_4_ nanoparticles were incorporated within MRBLEs, all MRBLEs in an Eppendorf tube could be attracted to one side of the tube by simply holding a magnet at the side of the tube (Fig. 4a and Movie S2).

To test whether MRBLE spectral codes can be resolved accurately even in the presence of 10% (v/v) Fe_3_O_4_ nanoparticle solution, we synthesized a small library of MRBLEs including a single Ln species. In prior encoding efforts, Tm has consistently been the least efficient Lns emitter^44^ due to the relatively low energy transfer efficiency between doped Tm^3+^ and VO_4_^3−^, resulting in weak blue emission^45^. To provide a particularly stringent test of potential coding capacity, we therefore synthesized magnetic MRBLEs containing the three lowest Tm/Eu levels used previously (see Fig. 2, codes 1, 2, and 19), mixed them, and attempted to resolve the three populations (Fig. 4b). All three levels were clearly distinguishable from one another, with a <0.01% probability of miscalling a code. Comparison of Tm/Eu cluster variance as a function of the median Tm/Eu level further established that variances were only ~2-fold higher in the presence of magnetic nanoparticles (Fig. 4c), suggesting that up to 1000-plex spectral code spaces remain possible. In addition, the measured median Tm/Eu levels changed only slightly (<5%) in the presence of magnetic nanoparticles, confirming the absence of nanoparticle-induced quenching. As with prior tests, magnetic MRBLEs were fairly monodisperse, with diameters of 51 ± 4 µm (Fig. 4d, mean ± standard deviation; CV = 7.8%).

### Exponential droplet splitting dramatically increases MRBLE production rates

**Fig. 5:**
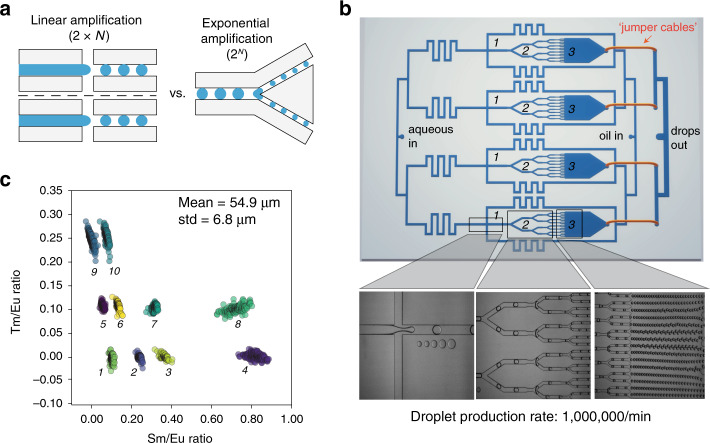
High-throughput MRBLEs 2.0 bead production via exponential droplet splitting. **a** Schematic demonstrating linear vs exponential droplet amplification. **b** Schematic of device for exponential droplet production including four flow-focusing nozzles for generating large droplets (*1*) and a series of 4 channel bifurcations (*2*) leading to a large channel for droplet collection (*3*) (top); representative images from these three device regions showing droplet formation, splitting, and collection (bottom). **c** Measured Tm/Eu and Sm/Eu ratios from a 10-code set produced via exponential droplet splitting with bead size indicated at top left

Leveraging MRBLEs for broad use requires the ability to rapidly and economically produce large numbers of beads. While increasing fluid flow rates can boost droplet production within a narrow range, large increases in flow rates drive a transition in droplet formation from dripping to turbulent jetting regimes, yielding very polydisperse beads. As an alternate approach, throughput can be increased by simply adding additional parallel flow focusers (FFs) to the ‘jumper cable’ synthesis device described above (Fig. 5a); however, this approach increases device size linearly with throughput (production rate scales as *N*, where *N* represents the number of FFs). To address this, droplet generation devices containing multiple Y-shaped splitters have been widely used to increase throughput^46,47^. Herein, we designed and fabricated an additional device that combined exponential droplet splitting with “jumper cables” to boost droplet production rates without increasing device area. In this device, a large droplet (~160 μm in lateral diameter and 50 μm in height) is initially split to form two smaller droplets upon encountering two bifurcated channels (Fig. 5a). These smaller droplets are subsequently split by more Y-junction structures, thus generating an exponential amplification of bead production (with rates increased by 2 ^*N*^, where *N* represents the number of splitting cycles) (Fig. 5b, c and Movie S3). As with the prior method, the produced droplets are routed to a single outlet via “jumper cables”, collected, and polymerized *en masse* via flood UV. The “jumper cables” route 4 smaller droplet-collecting chambers to 2 junction channels prior to collection from the droplet outlet, eliminating the need for glass inserts previously required to reduce deformation within large collection chambers^47^. For a device with 8 × 4 splitters (8 splitters in each pathway, 4 pathways in total), the measured droplet production rates averaged 10^6^/min at aqueous and oil flow rates of 1500 µL/h and 5400 µL/h, respectively, representing an ~3-fold increase over a linear production device with four FFs (Fig. 1b). The resultant droplets were slightly more polydisperse (CV = 12%), but spectral codes remained easily distinguished from one another (Fig. 5c**)**.

## Discussion

Developing multiplexed bead-based assays with the potential for broad translation and adoption requires the ability to produce large batches of spectrally encoded beads at low cost. Large bead batches make it possible to distribute beads widely for use in other labs during initial assay development and optimization and further minimize experimental noise resulting from batch-to-batch variation. Increasing the number of droplet generators within a device provides an obvious way to boost production rates, and this approach has been used successfully for a variety of different glass or PDMS droplet generation devices (including coflow nozzles^48^, T-junctions^49,50^, flow focusers^51–56^, and discontinuous step generators^49,50^). However, the need to route fluidic channels containing dispersed and continuous phases to and from many different droplet generating nozzles without channels intersecting one another presents fabrication challenges. In the past, this has been addressed by fabricating complex 3D wafer designs (for discontinuous step generators) or assembling devices from multiple PDMS layers with laser-cut or punched “vias” connecting the channels between layers (for T-junctions and flow focusers). Here, we adapt prior work that used tubing to connect different PDMS functional “modules”^57,58^ to create “jumper cables” that connect different channels within a single-layer device architecture. This strategy significantly reduces the fabrication complexity required to flexibly route channels in three dimensions while still minimizing fluidic dead volumes. Using this strategy, we demonstrated the ability to produce 150,000 MRBLEs/min (linear amplification) or 450,000 MRBLEs/min (exponential amplification), representing a pellet volume of ~1 mL/h or 3 mL/h and a 1000- to 3000-fold increase in throughput from prior work^14^.

The utility of spectrally encoded beads in downstream assays also depends critically on the ease with which analytes can be coupled to beads. Streptavidin- or antibody-conjugated hydrogels are often used to recruit and display biotinylated or epitope-tagged proteins, but these conjugations often rely on nonspecific absorption of biomolecules on the bead surface^9,10^, leading to the possibility of analyte loss and rebinding and consequent cross-contamination during long-term storage. The ability to covalently link molecules of interest to encoded beads bearing functional groups widely used in bioconjugation chemistry sidesteps this issue but can be difficult to implement with many commonly used fluorescently labeled bead matrices. Oligonucleotide-functionalized encoded hydrogels for nucleic acid quantification have been synthesized using continuous-flow lithography for years^59^, but the conjugation of “bait” molecules throughout the hydrogel mesh network^60^ can complicate measurements by dramatically increasing the time required for “bait”/”prey” binding interactions to reach equilibrium^61^. In addition, these approaches typically conjugate acrylated oligonucleotides and peptides, a modification that is more technically challenging for proteins^62^. QD-encoded microdroplets incorporating probe and target oligonucleotides and ratiometric QDs can solve the diffusion issue^63,64^, but this approach has been demonstrated only for DNA and again requires two-layer devices with automatic valving systems that increase the fabrication and instrumentation needed. Here, we demonstrated the ability to incorporate functional groups exclusively on the surface by simply generating aqueous polymer droplets in an oil stream containing functionalized comonomers bearing carboxyl or amino groups. This same approach could easily be extended to a variety of other functional groups^65^, including azides for downstream “click” chemistry, hydroxyl groups for the cyanogen bromide-activated coupling of proteins, hydrazide groups for oxidized carbohydrate proteins and chloromethyl groups for coupling NH_2_ groups in proteins or other biomolecules. In addition, this approach could facilitate the straightforward coupling of multiple molecules of interest to a single bead (e.g., proteins and oligonucleotides simultaneously) by adding multiple comonomers to the oil phase during droplet generation, facilitating the production of beads for single-cell profiling methods such as CITE-seq^66^ or ECCITE-seq^67^.

The simultaneous magnetic and spectral encoding strategy demonstrated here enhances the ability to remove a portion of molecules after coupling or synthesis to quantitatively assess bead loading or synthesis quality. As an example, biomolecules could be coupled or synthesized directly on a mixture of spectrally encoded beads and unencoded magnetic beads, making it possible to remove magnetic beads after reactions for elution and downstream characterization.

In future work, the simple encoded bead production and functionalization pipeline presented here could be integrated with various liquid handling robots and high-throughput droplet generation technologies for a variety of downstream commercial and translational applications. Lns, PEG-DA polymer, functionalized comonomers, and photoinitiators are all commercially available and relatively inexpensive, facilitating the development of low-cost assays. In addition, the ability to mix Lns and polymer before introducing solutions into devices for droplet generation makes it possible to automate this process for the production of large code sets using commercial liquid handling robots capable of mixing viscous solutions (e.g., LabCyte Echo or Bravo liquid handling systems). These mixed solutions could then be introduced into devices optimized for ultra-high-throughput droplet production via centrifugal methods^68–70^, in-air jetting droplet generators^71^, or glass-silicon chips recently shown to produce droplets at rates exceeding 5.5 billion droplets/min^51^. In this way, the production of Ln-encoded MRBLEs at the industrial scale could become feasible, opening up a wide range of new bead-based multiplexed assays.

## Materials and methods

### Device design, photolithography, and fabrication

All devices used in this paper were designed and fabricated via standard soft lithography protocols^72^. Briefly, microfluidic molding masters were created by (1) coating 4” test-grade silicon wafers (University Wafer, South Boston, MA) with a single layer of SU-8 2050 negative photoresist, (2) soft baking, (3) exposing this SU-8 layer to UV light passing through a printed transparency mask (designed in AutoCAD (Autodesk); printed at 50,000 dpi by Fineline Imaging), (4) postexposure baking, and (5) developing away uncured photoresist using SU-8 developer (Microchem Corp, Newton, MA) according to standard manufacturer instructions. These molding masters were then used to cast single-layer droplet generators composed of a 1:5 ratio of poly(dimethylsiloxane) crosslinker:base (PDMS, RTV 615, Momentive Performance Materials, Albany, NY), and these resultant devices were assembled as described in the Supplemental Methods. All holes were punched by a catheter punch (SYNEO, 0.025” ID × 0.035” OD, Part No: CR0350255N20R4) to fit the outer diameters of PEEK tubing (ZEUS, 0.010” ID × 0.020” OD) and steel blunt pins (New England Small Tube, Part No: NE-1310-02). All design files and detailed protocols for the molding master and device fabrication are available as Supplementary Files and in an associated OSF repository (https://osf.io/jvnpc/).

### Bead synthesis

Mixtures of PEG-DA polymer, Lns, and LAP photoinitiator were prepared largely as described previously^14^. In general, premixed formulas (250 μL in total) were generated by varying the ratios of three monomer master mixtures, each containing different Lns. All aqueous master mixtures contained purified water with 21.4% v/v PEG-DA (Sigma-Aldrich, average Mn 700) and 5% v/v YVO_4_:Eu (50 mg/mL). The “Dy”, “Sm” and “Tm” master mixtures also contained 16.3% v/v YVO_4_:Dy (50 mg/mL), 16.3% v/v YVO_4_:Sm (50 mg/mL) and 16.3% v/v YVO_4_:Tm (50 mg/mL), respectively. Each formula was prepared based on the reference ratio listed in Table S1. Directly prior to the injection of polymer solutions into the device, we added 3% v/v LAP (Sigma-Aldrich, 39.2 mg/mL in DI water) photoinitiator. The produced Ln/polymer droplets were collected through Tygon tubing into a 24-well plate (Thermo Fisher Scientific). To prevent premature evaporation of HFE7500 and resultant droplet breakage during initial droplet production, we filled each well with ~80 μL of oil solution prior to droplet generation. To polymerize these Ln/polymer droplets into solid MRBLE beads, we flood-exposed wells to UV light (IntelliRay, UV0338) for 2 min at 100% amplitude (7” away from the lamp, power = ~50–60 mW/cm^2^). We recommend direct UV polymerization after the production of every two codes to reduce any potential droplet breakage. After polymerization, we washed the beads with 2 mL of dimethylformamide (DMF, Thermo Fisher Scientific) for 20 s, with 2 mL of dichloromethane (DCM, Thermo Fisher Scientific) for 10 s and with 2 mL of methanol (Thermo Fisher Scientific) for 20 s and then resuspended them in either 1 mL of 1X phosphate-buffered saline (PBS) (Thermo Fisher Scientific) with 0.01% (v/v) Tween-20 (Sigma-Aldrich) (PBST) for the aqueous EDC chemistry and biotinylation of NH_2_-MRBLEs or in dimethyl sulfoxide (DMSO, Thermo Fisher Scientific) for oligonucleotide conjugation. To make magnetic MRBLEs, we added 10% v/v of the Fe_3_O_4_ nanoparticle solution (150 mg/mL) to each recipe. Detailed protocols for the synthesis of Lns and magnetic nanoparticles are provided in the Supplemental Methods.

### Oligonucleotide conjugation

To prepare for oligonucleotide conjugation, beads were rinsed three times with 200 µL of DMSO by spinning down the beads into a pellet and removing the supernatant. After the final spin, we resuspended the beads in DMSO at a concentration of 10,000 beads per 170 µL of reaction volume. We assembled the “conjugation reaction” by combining 20 µL of EDC (Sigma-Aldrich) from a 300 mM stock solution prepared by dissolving 58 mg EDC in 1 mL of DMSO; 20 µL of 1-hydroxy-7-azabenzotriazole (HOAT, Sigma-Aldrich) from a 60 mM solution prepared by dissolving 9.2 mg of HOAT in 1 mL of DMSO; and 20 µL of diisopropylethylamine (DIPEA, Sigma-Aldrich) from a 300 mM solution prepared by adding 32 µL of DIPEA to 968 µL of DMSO. We then incubated beads in this EDC, HOAT, and DIPEA solution for 15 min on a shaker (or rotator at 15 rpm) to ensure adequate mixing. After 15 min, we added 10 µL of a 100 µM stock solution of oligonucleotides modified at the 5’ end with amines and a carbon spacer (the “5AmMC12” modification from Integrated DNA Technologies) and modified at the 3’ end with Alexa 647 dye and an additional 20 µL each of EDC, HOAT, and DIPEA^73^. We then incubated the reaction at room temperature for 16 h on a shaker. After this incubation, we neutralized the conjugation reaction by adding 50 µL of 500 mM ethanolamine solution (prepared by adding 30.2 µL ethanolamine (Sigma-Aldrich) to 968 µL DMSO) to each reaction and incubating for 1 h. Following conjugation, we washed beads three times with 200 µL of PBS containing 0.1% (v/v) Tween 20. We then combined all beads into a single Eppendorf tube, resuspended them at 100 beads/µL in PBS with 0.1% (v/v) Tween 20, and stored them at 4 °C.

### Aqueous EDC chemistry

For streptavidin-coated MRBLEs, we washed 150 μL of carboxy MRBLEs and resuspended them in 200 μL of MES buffer (pH = 4.5) supplemented with 0.01% (v/v) Tween 20. Next, we added 200 μL of freshly made EDC solution (2% w/v, corresponding to 10 mg of EDC in 500 μL MES buffer) into the bead solution and incubated the entire reaction for 3.5 h at room temperature on a rotator. The O-acylisourea intermediate on the beads is unstable in aqueous solutions; thus, the bead mixture was subsequently immediately washed with 1 mL of borate buffer (pH = 8.5) supplemented with 0.01% (v/v) Tween 20 (coupling buffer) and resuspended in 400 μL of coupling buffer. To conjugate streptavidin, we added 16 μL of 1 mg/mL streptavidin (Sigma-Aldrich, dissolved in PBS) in borate buffer to the mixture and rotated the whole slurry overnight at 4 °C. After incubation, we quenched the reaction by adding 10 μL of 0.25 M ethanolamine in borate buffer and incubating the solution on a rotator for 30 min at 4 °C. The final product was washed three times and resuspended in 200 μL of PBST buffer for further use. To test the conjugation efficiency, we incubated 20 μL of streptavidin MRBLEs with 0.5 μL of FITC-biotin (Thermo Fisher Scientific, 10 mg/mL in DMSO) for 1 h on an end over end rotator at 30 rpm, washed with 1 mL of PBST three times, and then imaged. In our experience, beads can be stored at 4 °C for ~6 months without a loss of streptavidin binding efficiency.

### Biotinylation of NH2-MRBLEs

MRBLEs coated with amine groups using 0.09% (v/v) pent-4-enylamine were stored in PBST and extensively washed with DCM, methanol, and DMF prior to biotin conjugation. For each conjugation reaction, we combined ~10,000 beads with 39 mg of biotin, 24 µL of N,N’-diisopropylcarbodiimide (DIC, Sigma-Aldrich) and 56 µL of DIPEA in 400 μL of DMF twice overnight, rotating at room temperature. After conjugation, we washed beads serially with 1 mL each of DMF, methanol, DCM, DMF, water and PBST. After washing, we passivated ~2000 beads with 5% BSA PBST for 1 h at room temperature on a rotator at 30 rpm; the other ~8000 beads were stored at 4 °C. We then exchanged the 5% BSA PBST solution to 2% BSA in PBST by washing and resuspension, added 1 µL of 1 mg/mL DyLight 650-tagged streptavidin (Abcam), and incubated for 30 min at 4 °C on a rotator at 30 rpm. Finally, we washed all beads three times with PBST and imaged them as described below.

### Bead imaging and data analysis

Bead imaging was performed on a Nikon Ti microscope with a custom UV transilluminator largely as described previously^13^. Briefly, beads were excited using a Xenon arc lamp (Lambda LS, Sutter Instruments, Novato, CA) with an automated filter wheel (Lambda 10-2, Sutter Instruments, Novato, CA) containing filters designed to pass 292 nm light (a 292/27 bandpass excitation filter (Semrock, Rochester, NY) paired with UG11 absorptive glass (Newport, Irvine, CA) and were imaged for lanthanide emission at 9 distinct wavelengths using nine emission filters (435/40, 474/10, 536/40, 546/6, 572/15, 620/14, 630/92, 650/13, and 780/20 nm) and captured images using an sCMOS camera (Andor) (Andor Technology plc., Belfast, Northern Ireland). We then extracted the most probable Ln ratios associated with each bead via linear unmixing relative to a series of Ln reference spectra^44^. The binding of Alexa 647-labeled DNA oligonucleotides, FITC-biotin, and DyLight 650-labeled streptavidin was detected via excitation using a SOLA light engine (380 nm -680 nm, Lumencor, Beaverton, OR) and visualized using Cy5, eGFP, and Cy5 filter cube sets, respectively. For imaging beads with more than 2 codes, we recommend dyes excited at higher wavelengths (e.g., Cy5, Alexa 647, and DyLight 650), as fluorescent dyes excited at shorter wavelengths (e.g., FITC) may be excited by deep-UV light and emit light in the Ln channels, complicating decoding. However, emission from the Ln-encoded beads under SOLA light engine excitation was not observed in the fluorescent dye channels used here, as shown in Figure S9.

## Supplementary information

Editorial Summary

SI figures and SI methods_clean version

Movie S1

Movie S2

Movie S3
