# Supplementary material for: MRBLES 2.0: High-throughput generation of chemically functionalized spectrally and magnetically encoded hydrogel beads using a simple single-layer microfluidic device
Source: Microsyst Nanoeng. 2020 Nov 30;6:109. doi: 10.1038/s41378-020-00220-3 (PMC7704393; doi:10.1038/s41378-020-00220-3)
Supplement: Supplementary file 1 — Editorial Summary [file 41378_2020_220_MOESM1_ESM.docx]

# *Microsystems & Nanoengineering*

**Microfluidics: high-throughput production of spectrally encoded hydrogel beads for bioassays**

Spectrally encoded beads provide a convenient platform for multiplexed bioassays, offering fast binding kinetics and many replicates per assay. A recently developed technology, MRBLEs, spectrally encodes hydrogel beads via the ratiometric incorporation of lanthanide nanophosphors. In the present paper, a team from Stanford University led by Polly Fordyce reports a dramatically simplified method for producing MRBLEs beads bearing various functional groups for downstream chemical coupling or on-bead synthesis. Using a ‘jumper cable’ tubing strategy, they route microfluidic channels in 3D without a need for complex fabrication techniques to create multi-nozzle droplet generators. Using these simple single-layer microfluidic devices, they create beads with 48 unique spectral codes bearing carboxyl and amine groups for downstream coupling with over 1000-fold increase in throughput. Finally, they demonstrate that MRBLEs can be simultaneously spectrally and magnetically encoded.

Related article manuscript number: MICRONANO-01345R

Article title: MRBLEs 2.0: High-throughput generation of chemically functionalized spectrally and magnetically-encoded hydrogel beads using a simple single-layer microfluidic device

Corresponding author and affiliation/s: Polly Fordyce, Stanford University, United States

**About your Editorial Summary — please read**

**Before approving this Editorial Summary, please carefully check that (1) the summary text lists the correct author(s) and (2) the spelling and order of all author names and affiliations are correct.**

This **Editorial Summary** is based on your manuscript that was recently accepted for publication in *Microsystems & Nanoengineering*. It provides a non-specialist audience with a synopsis of your key research outcomes and conclusions. This value-added service provided by Springer Nature is designed to raise interest in your research across the broader community.

Springer Nature will publish the summary on the journal’s website, and it will be freely available under a under the CC BY licence (Creative Commons Attribution v4.0 International Licence) (see the journal website for details). We encourage you to re-use the summary to bring attention to your research; for example, you can host it on your own website and share it via social-networking platforms. Please attribute the summary to *Microsystems & Nanoengineering* and your article (e.g. by providing a link to your article) and do not make derivatives.

Please note that to maximise the usefulness of these summaries they must follow several stringent guidelines:
-- Spelling, punctuation and style are set according to *Nature* editorial guidelines. As this summary is aimed at non-expert readers, some concepts and technical terms will be simplified.
-- Total length must be no more than 135 words. It is likely that not all points in the paper will be covered.
-- The first sentence must be no more than 280 characters, including spaces, to allow use on microblogging sites.
-- The headline must consist of a brief generic subject identifier followed by a short description. No more than 10 words in total.

Please contact the editorial office ([mems_nano@mail.ie.ac.cn](mailto:mems_nano@mail.ie.ac.cn)) immediately with corrections should you find any factual errors in this Editorial Summary.
